# TopControl: A Tool to Prioritize Candidate Disease-associated Genes based on Topological Network Features

**DOI:** 10.1038/s41598-019-55954-6

**Published:** 2019-12-19

**Authors:** Maryam Nazarieh, Volkhard Helms

**Affiliations:** 1 Graduate School of Computer Science, Saarland University, Saarbruecken, Germany; 2 Center for Bioinformatics, Saarland University, Saarbruecken, Germany

**Keywords:** Network topology, Gene regulation

## Abstract

Putative disease-associated genes are often identified among those genes that are differentially expressed in disease and in normal conditions. This strategy typically yields thousands of genes. Gene prioritizing schemes boost the power of identifying the most promising disease-associated genes among such a set of candidates. We introduce here a novel system for prioritizing genes where a TF-miRNA co-regulatory network is constructed for the set of genes, while the ranks of the candidates are determined by topological and biological factors. For datasets on breast invasive carcinoma and liver hepatocellular carcinoma this novel prioritization technique identified a significant portion of known disease-associated genes and suggested new candidates which can be investigated later as putative disease-associated genes.

## Introduction

Gene expression analysis is the basis for identifying differentially expressed (DE) genes between two conditions. There are usually hundreds to thousands of DE genes between, e.g., tumor and matched normal samples^1,2^. Candidate gene prioritization helps experimentalists to focus their follow-up experiments on the most promising candidates among these. A typical feature of candidate disease genes is the amount of de-regulation (log fold chance of expression, LFC) between disease and normal conditions. Another criterion is whether they belong to the highly connected (hub) genes either in a gene-regulatory network context or in the related protein-protein interaction network.

Prioritization tools typically produce their outputs either by filtering the candidates into smaller subsets or by ranking the candidates from the most promising to the least promising ones. Currently, ranking methods based on network analysis require prior knowledge about the disease at hand. For example, they combine interaction networks with functional annotations to select disease-associated candidates such as ToppGene^3^. The tool ToppNet^4^ takes a different approach and ranks the candidate genes based on topological features in a related protein-protein interaction (PPI) network. The tool utilizes three algorithms mainly developed for social networks to prioritize candidate genes.

Other ranking approaches use text mining techniques to select the candidates related to a disease by retrieving related documents from literature focusing on certain keywords^5^. Since many prioritization methods require access to multiple databases, they are available mostly in the form of web services, e.g, Endeavor^6^. The tool integrates 75 data sources for six species including *H*. *sapiens* and *M*. *musculus*. After selecting one of the six available species, a set of genes is provided by the user which are associated to the biological process of interest. This set is used for training a model. Users can select among a variety of data sources for prioritizing the list of candidates. Finally, an ordered list of candidates with the most promising ones on top is the output. Unlike Endeavor, NetworkPrioritizer^7^ utilizes the central nodes of a network mainly based on betweenness and closeness as seed nodes. It aggregates multiple node rankings derived according to the distance to central nodes to prioritize the candidates. However, it is not clear whether high centrality genes exercise a full control over the underlying network. FocusHeuristics aims to combine the static knowledge on the PPI network with the dynamics of gene expression for the gene ranking^8^. The software aggregates three features that are derived from gene expression data and the biological network, namely fold change, the differential link score and the interaction link score. Then the tool prunes the network to those nodes that exceed at least one of the predefined thresholds of the mentioned features. Therefore, the results vary when different thresholds are used. Gene Ranker, which is a semi-supervised approach, incorporates a weighted gene co-expression network into a PPI network to construct a disease-specific network^9^. This is mainly performed by adding the gene expression data of patients to the PPI network. This semi-supervised approach uses the known disease-associated genes to identify and prioritize the set of unknown genes that are related to a specific disease.

In previous work, we demonstrated the ability of minimum dominating set (MDS) and minimum connected dominating set (MCDS) in capturing a significant portion of known drug target genes in a differential regulatory network for breast cancer^10^. Moreover, we showed that a MCDS had high overlap with betweenness central nodes in the network, whereas the closeness centrality was more dominant in the MDS for the mouse ESC network^10^.

**Figure 1:**
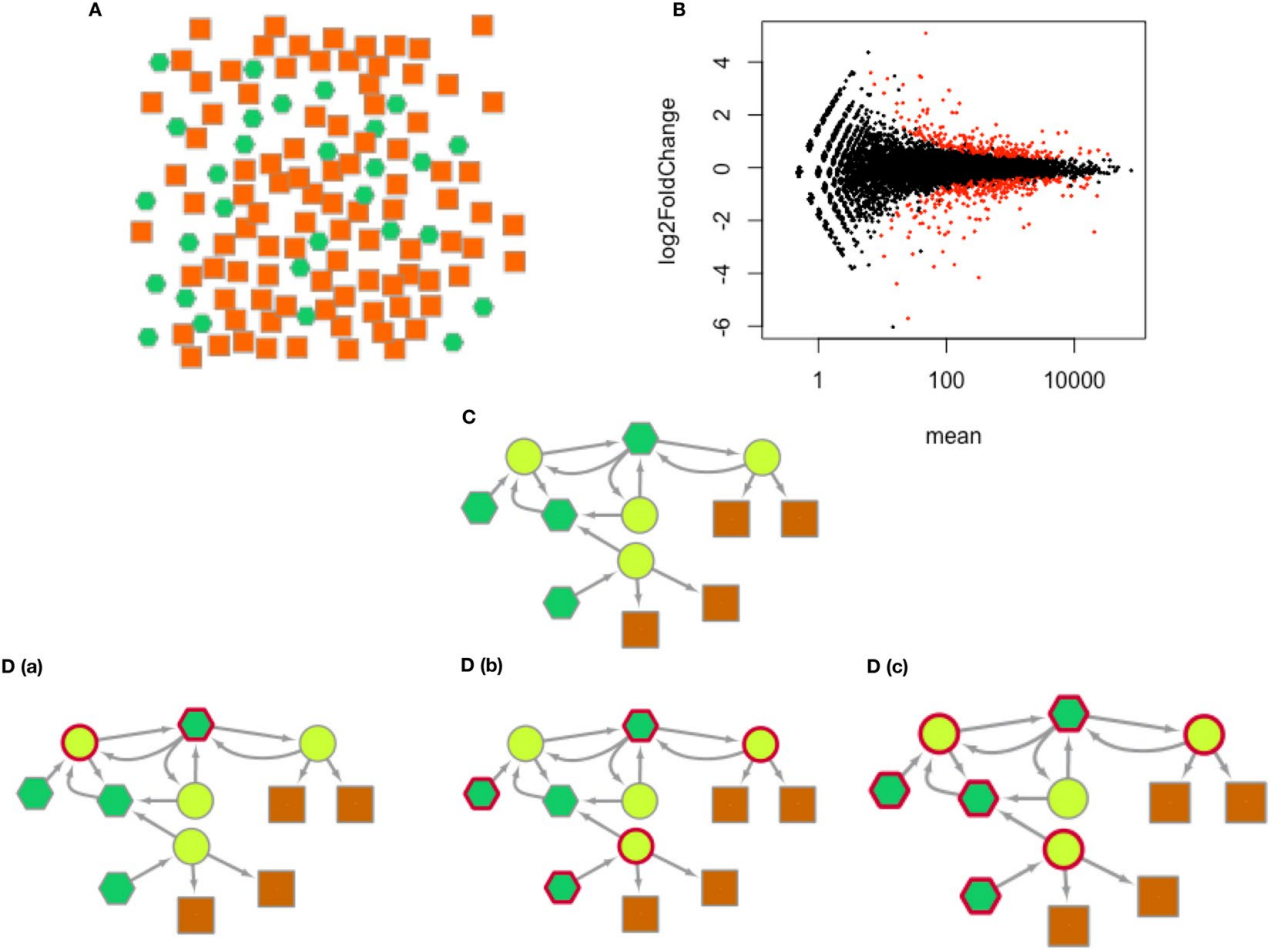
A graphical representation that illustrates the TopControl method. (**A**) In the first layer all the genes and TFs are considered as potential candidates. (**B**) In the second layer, candidates are prioritized if they are significantly differentially expressed between two conditions. Here, the red points are the significant DE genes determined by DESeq. (**C**) The third layer contains DE genes and miRNAs identified by TFmiR that execute 1 out of 4 types of considered interactions (see text). The red circle borders mark the hubs, MDS and MCDS of the underlying network in panels (D(a–c)), respectively.

Here, we propose the novel candidate gene prioritization method TopControl based on systematic analysis of DE genes between tumor and normal conditions. TopControl does not rely on any prior knowledge for prioritization of candidates. This means that no seed genes need to be provided by the user. Initially, DE genes are filtered out to yield a set of candidates which form a network. The network candidates are prioritized based on network topological features. We assume that the priority of the candidates by which we mean the relevance to a disease in a differential co-regulatory network increases when they belong to either MDS, MCDS or hub sets. Since significant DE genes with high fold changes are usually considered as top candidates^11^, the set of candidates with equal topological priorities are sorted based on the log_2_(fold change) (LFC) of expression. The concept of TopControl is visualized for a small network in Fig. 1. It turned out that topological criteria (hub degree nodes and dominating nodes) appear more successful for the two datasets studied here in identifying candidate disease genes than LFC alone.

## Results

We tested TopControl on processed RNA-Seq data taken from The Cancer Genome Atlas (https://cancergenome.nih.gov) for matched tumor and normal samples of liver hepatocellular carcinoma (LIHC) and breast invasive carcinoma (BRCA) datasets. They were downloaded in June, 2015.

### LIHC dataset

**Figure 2:**
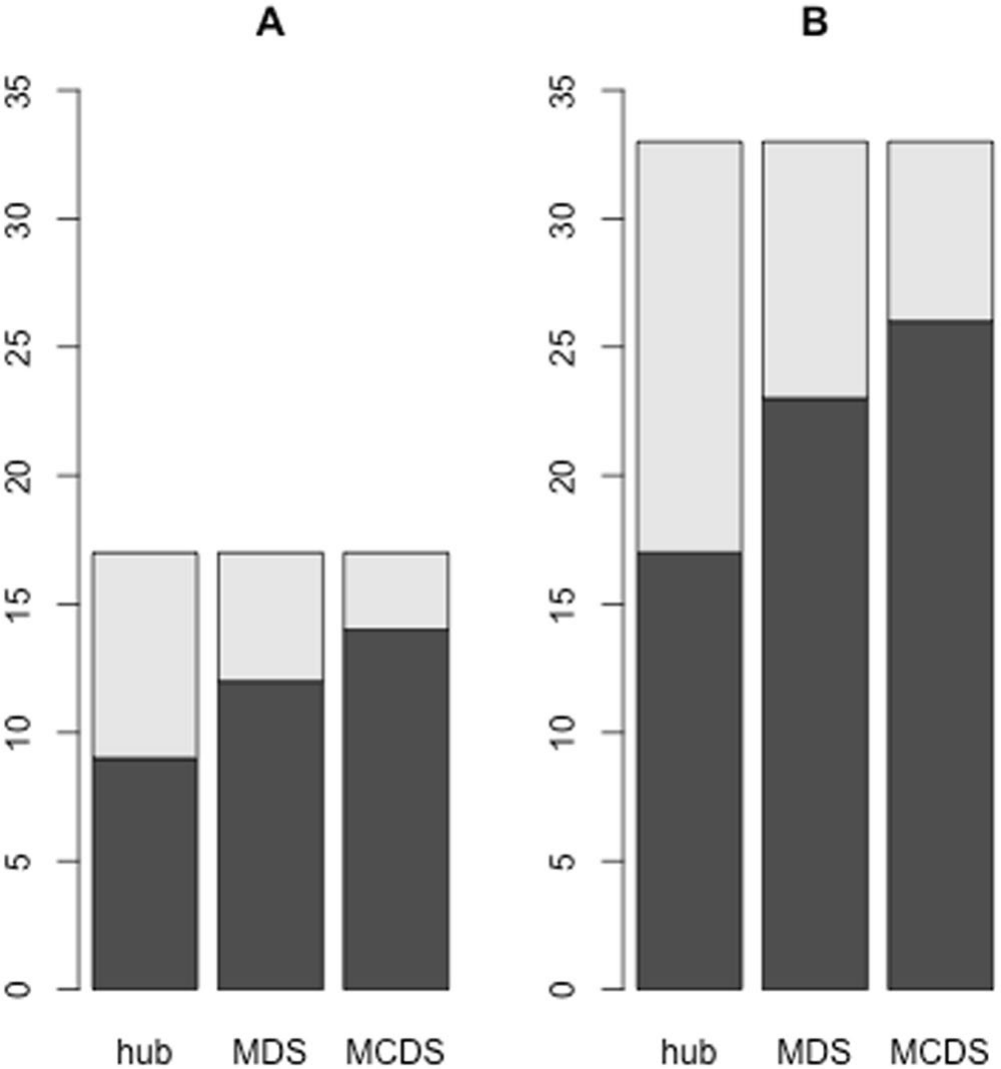
Overlap of disease-associated genes and miRNAs with the topological features of TopControl. The black portions mark the overlap of disease-associated genes and miRNAs with hubs, MDS and MCDS of the corresponding co-regulatory networks derived from **A**) LIHC and **B**) BRCA datasets. The gray portions show the total candidates in the fourth layer of TopControl that are associated with the related disease.

The LIHC dataset consisting of transcripts as level 3 Illumina HiSeq-RNASeq V2 data of 20501 genes for 100 matching tumor and normal samples was used as input to the DESeq method^12^. DESeq identified 3872 significant DE genes with adjusted *p*-values below 0.05. This set of DE genes was then provided as an input to the TFmiR web server^13^, setting the *p*-value threshold to 0.05 and selecting experimental resources. We have recently shown^14^ that TFmiR results are robust toward different methods used to identify differentially expressed genes. We showed this on the example of the same RNAseq data sets for LIHC and BRCA that were used here. TFmiR constructed a TF-miRNA differential co-regulatory network with 270 genes, 5 miRNAs, and 383 regulatory interactions. Twenty-eight hub-degree nodes (top 10%) were obtained from the hotspot section of the web server. MDS and MCDS contained 61 and 68 genes and miRNAs, respectively. The union of hubs, MDS and MCDS contains 82 distinct genes and miRNAs, see Table S1. Figure 2 shows the overlap of known disease-associated genes and miRNAs with the identified hubs, MDS and MCDS, respectively. MCDS contains the largest number of candidates among them.

Table 1 shows the set of genes and miRNAs associated with hepatocellular carcinoma (HCC) in the TFmiR complete network with the corresponding scores assigned by TopControl. Seventeen out of 33 genes and miRNAs associated with HCC (according to the databases DisGeNET^15^ and HMDD database^16^) in the network belong to the set chosen by TopControl with a score greater than zero. Table S1 shows the list of candidates in the fourth layer. Considering the known disease-associated genes and miRNAs in DisGeNET and HMDD databases, the identified genes and miRNAs in the fourth layer led to *sensitivity* = 52%, *specificity* = 73% and *accuracy* = 71%. The significance of the union of genes and miRNAs selected by the respective methods gave a *p*-value of 0.004 (hypergeometric test) with *p*-values of (0.002, 0.035, 0.013), respectively for each individual set of hub, MDS, and MCDS when considering all nodes in the constructed network as background.

**Table 1:** TopControl-assigned scores of 17 disease-associated genes and miRNAs reported in DisGeNET and HMDD databases that belong to the full co-regulatory network for the DE genes of the LIHC dataset constructed by TFmiR.

gene	D	hub	mds	mcds	core	LFC
E2F1	25	1	1	1	3	3.76
ESR1	9	1	1	1	3	2.19
JUN	33	1	1	1	3	1.39
MYC	18	1	1	1	3	1.07
hsa-let-7b	47	1	1	1	3	—
hsa-mir-29a	21	1	1	1	3	—
FOXM1	3	0	1	1	2	3.69
FOS	23	1	0	1	2	2.93
CEBPD	1	0	1	1	2	1.3
PDGFB	1	0	1	1	2	1.11
SREBF2	3	0	1	1	2	0.68
NFE2L2	4	0	1	1	2	0.66
TERT	6	1	0	0	1	9.17
RRM2	2	0	0	1	1	2.98
AR	3	0	1	0	1	1
HTATIP2	2	0	0	1	1	0.68
CCND1	12	1	0	0	1	0.67

The candidates with the highest scores are at the top of the table and sorted by LFC in case of a similar score. D stands for the degree of the node and LFC for log_2_(fold change) of expression.

Enrichment analysis for these sets (hubs, MDS, MCDS) yielded the enriched GO terms and KEGG pathways listed in Tables S2, S3 and S4. These three sets shared several GO terms such as GO:0051726, GO:0008285 and GO:0042493 for regulation of cell cycle, negative regulation of cell proliferation and response to drugs, respectively. Moreover, MDS and MCDS shared the GO:0010941 term (regulation of cell death).

Genes and miRNAs selected by all three criteria constitute the top-most candidates in the fifth layer. Six out of 18 proposed genes and miRNAs in the fifth layer were reported in DisGeNET and HMDD. By way of construction, all considered genes are differentially expressed between normal and tumor conditions. In fact, all 16 genes are significantly expressed. Moreover, the other 12 candidates not mentioned in the above-mentioned databases were significantly expressed in hepatocellular carcinoma tissue according to LiverWiki^17^.

### BRCA dataset

Next, the breast invasive carcinoma (BRCA) dataset with transcripts as level 3 Illumina HiSeq-RNASeq V2 data for 20501 genes and 226 matching tumor and normal samples was used as input to the DESeq method^12^. The later version of the dataset was explained and applied in PPIXpress in^18^. A total of 5231 significant DE genes with adjusted *p*-values below 0.05 were identified by DESeq. The set of DE genes was provided as an input to the TFmiR web server, by setting the *p*-value threshold to 0.05 and selecting experimental resources. TFmiR constructed a differential co-regulatory network with 463 nodes (457 genes and 6 miRNAs) and 696 regulatory interactions. For this differential co-regulatory network, 97 and 113 genes and miRNAs were the results of MDS and MCDS, respectively. Together with the 47 hub-degree nodes, this led to a total of 140 distinct genes and miRNAs.

Table 2 shows the set of genes and miRNAs associated with breast neoplasms (BN) with the corresponding TopControl-assigned scores. Thirty-three out of 50 genes and miRNAs associated with BN (according to the databases DisGeNET^15^ and HMDD^16^) in the network belong to the set selected by TopControl. As shown in Fig. 2B, the MCDS contains the highest proportion of known disease-associated candidates. Considering the candidates in the fourth layer shown in Table S5 led to *sensitivity* = 66%, *specificity* = 74% and *accuracy* = 73%, respectively. The significance of the overlap was measured using the hypergeometric test with *p*-value of $3.15\ast {10}^{-8}$ with (6.68e-7, 2e-50, 6e-6) for the individual sets (hub, MDS, MCDS) by considering all nodes in the constructed co-regulatory network as background.

**Table 2:** TopControl-assigned scores for 33 disease-associated genes and miRNAs reported in DisGeNET and HMDD databases that belong to the constructed TFmiR complete network for the BRCA dataset, see Table 1.

gene	D	hub	mds	mcds	score	LFC
ESR2	7	1	1	1	3	2.58
FOS	20	1	1	1	3	2.47
E2F1	25	1	1	1	3	2.34
ESR1	19	1	1	1	3	1.79
JUN	45	1	1	1	3	1.6
STAT5A	7	1	1	1	3	1.6
ETS2	6	1	1	1	3	1.15
TFAP2A	24	1	1	1	3	0.9
hsa-mir-21	44	1	1	1	3	—
hsa-mir-146a	31	1	1	1	3	—
WT1	2	0	1	1	2	5.39
IFNB1	9	1	0	1	2	4.13
FOXM1	1	0	1	1	2	3.54
KIT	6	1	0	1	2	2.79
IL6	7	1	0	1	2	2.76
FOXA1	3	0	1	1	2	1.87
RARB	6	1	1	0	2	1.1
TRERF1	1	0	1	1	2	1.08
HEY2	1	0	1	1	2	1.06
MEIS1	1	0	1	1	2	1.01
NR2F6	2	0	1	1	2	1
KRAS	1	0	1	1	2	0.83
AR	5	0	1	1	2	0.63
ERBB2	6	1	0	0	1	1.89
BRCA2	4	0	0	1	1	1.81
AFP	1	0	1	0	1	1.43
EGFR	8	1	0	0	1	1.4
PDGFA	2	0	0	1	1	1.33
BRCA1	5	0	0	1	1	1.24
PARP1	1	0	1	0	1	1.2
CCND1	15	1	0	0	1	1.01
PGR	5	0	0	1	1	0.87
ZEB1	1	0	1	0	1	0.72

Enrichment analysis for these sets (hub, MDS, MCDS) identified the enriched biological process GO terms and KEGG pathways listed in Tables S6, S7 and S8. These three sets shared some enriched GO terms such as GO:0008285 and GO:0042493 for negative regulation of cell proliferation and response to drugs, respectively. The three sets also shared several KEGG pathways related to different cancers. Moreover, MDS and MCDS shared several terms related to cell cycle and cell differentiation. The top-most candidates suggested by TopControl are the ones with the highest scores. Some of them such as ESR2, FOS, E2F1, ESR1, JUN, STAT5A, ETS2, TFAP2A, hsa-mir-146a and hsa-mir-21 are disease-associated genes based on the DisGeNET and HMDD databases. Other candidates such as EGR1, RUNX2, STAT1, TRAP2, IRF1 and USF1 have been experimentally validated as drug targets, metastasis promoter or tumor growth enhancers, see^19–24^.

### Common candidates among related diseases

**Figure 3:**
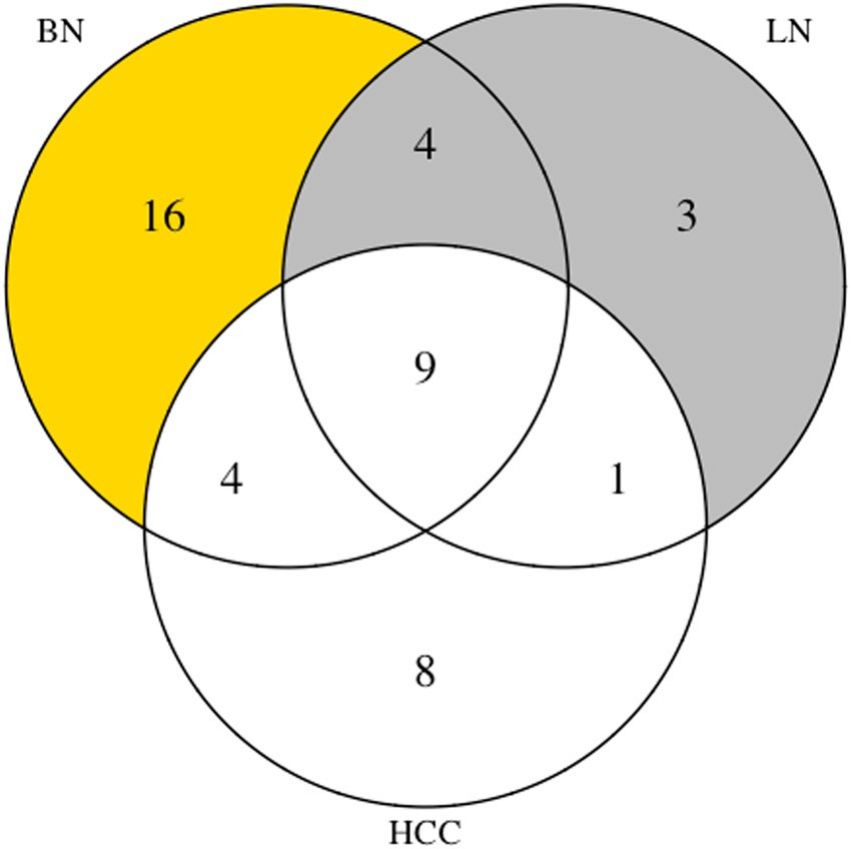
Number of experimentally-validated disease-associated genes and miRNAs for BN, HCC and LN selected by TopControl. The figure also shows the number of common candidates among them.

Breast cancer can spread to other organs in the body such as lung and liver^25^. To check the potential of the constructed co-regulatory network to reflect co-association with other diseases, we considered the set of disease-associated genes and miRNAs that were among hubs, MDS and MCDS of the differential co-regulatory network for breast cancer. The network included 39 experimentally validated HCC-associated genes and miRNAs. Twenty-two of them were among the hubs, MDS and MCDS of the network. In case of lung neoplasms (LN), 17 out of 28 lung cancer-associated genes and miRNAs were among the candidates in the fourth layer. The overlap among the set of hubs, MDS and MCDS that were experimentally-validated for BN with both HCC and LN was 13 out of 33, whereby the intersection between all of them was 9, see Fig. 3. Table 3 lists the common genes and miRNAs between experimentally-validated BN with HCC and with LN. Eight and 7 out of the 25 top-most candidates in the network were also experimentally-validated genes and miRNAs for HCC and LN. FOS, ESR1, JUN, hsa-mir-146a and hsa-mir-21 were common among all the experimentally-validated genes and miRNAs in the fifth layer.

**Table 3:** Disease-associated genes and miRNAs that are common between BN and HCC or between BN and LN.

Disease	Common genes
HCC	FOS, E2F1, ESR1, JUN, hsa-mir-146a, hsa-mir-21, FOXM1, IL6, KRAS, AR, AFP, EGFR, CCND1
LN	FOS, ESR1, JUN, STAT5A, hsa-mir-146a, hsa-mir-21, WT1, FOXM1, RARB, KRAS, ERBB2, EGFR, CCND1

### Comparison of TopControl with Endeavor

**Figure 4:**
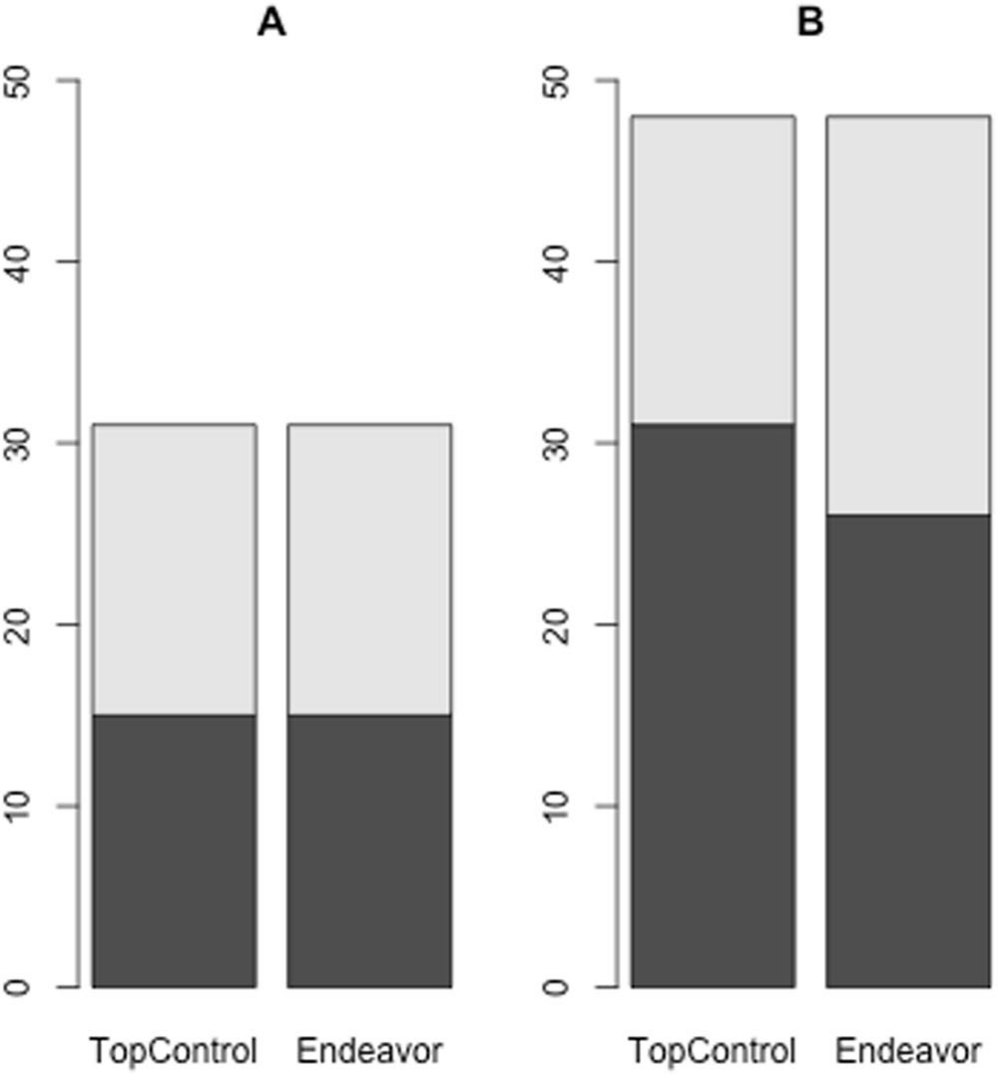
Comparison of identified disease-associated genes between TopControl and Endeavor for **A**) the LIHC and **B**) BRCA datasets. The black areas mark the overlap of disease-associated genes identified by the tools. The gray portions show the total number of disease-associated genes in the networks without considering miRNAs.

To the best of our knowledge, there is currently no other tool available that works without relying on prior knowledge. Hence, we compared the results of TopControl with those from Endeavor^6^, whereby we provided this tool with the minimum number of required information (a single training candidate). For this, the sets of DE genes for the LIHC and BRCA datasets derived with DESeq were given as input to Endeavor, separately. We selected the same number of genes as obtained by TopControl from the top selected genes by Endeavor based on the *p*-value that stands for the significance of a combination of rankings. In this comparison, we detained the set of miRNAs from the lists of top candidates obtained by TopControl as Endeavor does not consider miRNAs. For HCC, TP73 which is known to be associated with HCC, was used as a training candidate. For BN we selected ESR1 that is a known BN-associated gene for the training phase of Endeavor. We selected all data sources to build models and prioritize the candidates from Endeavor. DisGeNET was used for the evaluation of the results. For the case of hepatocellular carcinoma, both methods performed equally well by detecting 15 related disease-associated genes among 77 top candidates. The overlap among the identified disease-associated genes comprises seven genes (E2F1, MYC, JUN, CCND1, ESR1, TERT, SREBF2). In the case of breast neoplasms, TopControl outperformed the minimum-information version of Endeavor with detecting 31 compared to 26 related disease-associated genes out of 134, see Fig. 4. This led to an overlap of 20 genes including ESR2, PGR, AR, JUN, CCND1, RARB, NR2F6, EGFR, FOXA1, FOS, ERBB2, E2F1, WT1, BRCA1, KRAS, TFAP2A, ZEB1, STAT5A, TRERF1, and PARP1.

### How TopControl works

**Figure 5:**
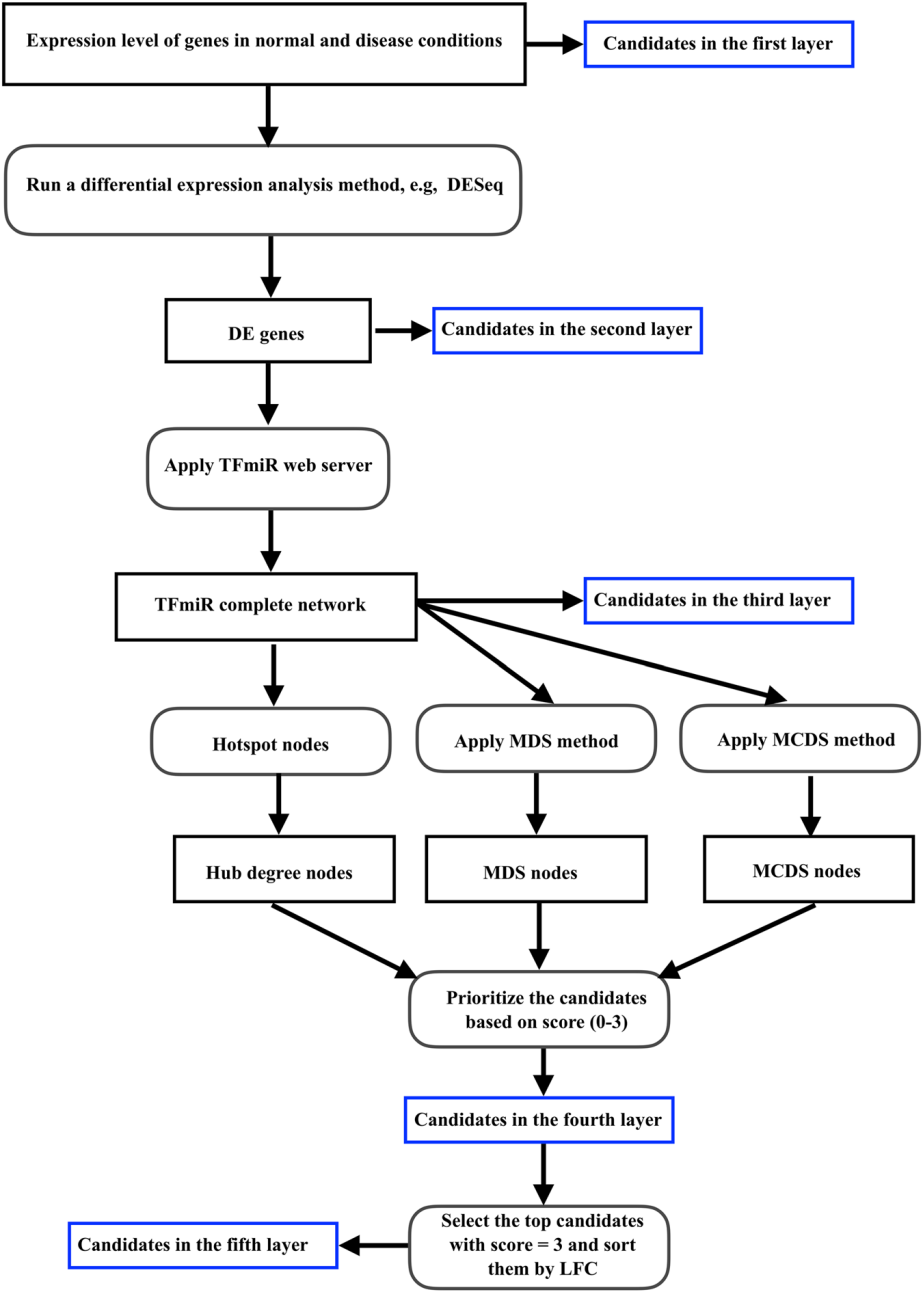
TopControl Workflow. TopControl integrates three sets of hub, MDS and MCDS genes as candidates in the fourth layer. The set of genes and miRNAs that are selected by all the above-mentioned methods make up the candidates in the fifth layer.

TopControl is a program written in the R programming language that is available at (https://github.com/maryamNazarieh/TopControl). To run TopControl as shown in Fig. 5, the user needs to upload a set of DE genes as an input to the TFmiR web server^13^. No disease is required to be selected. TFmiR then constructs a differential co-regulatory network and outputs the set of hub degree genes and miRNAs for the network. MDS and MCDS can be obtained for the co-regulatory network by executing the related tools^10^. The R program integrates and prioritizes the three sets of hubs, MDS and MCDS as input and returns the candidates in the fourth and fifth layers provided as output. Moreover, it returns a set of experimentally-validated candidates in the fourth layer which are reported in the databases DisGeNET^15^ and HMDD^16^.

## Discussion and Conclusion

This work introduced the tool TopControl as a novel prioritization method based on topological and biological factors to propose a set of disease-associated candidate genes and miRNAs. TopControl differs from other gene prioritization tools mainly in that it does not rely on any prior knowledge to train and build models for the prediction of disease-associated genes. The tool takes a systematic approach to suggest the set of most-relevant candidates to a certain disease. TopControl enhances the relevance of the candidates to a specific disease if they are either members of the hub set, MDS or MCDS. Although both MDS and MCDS consider a set of nodes that can control the state of a GRN through direct regulation of their target genes, only MCDS ensures that network controllability is achieved by a dominating pathway in the underlying network. The most promising candidates suggested by this tool are the ones that are selected by all the aforementioned methods. Among these, TopControl considers the LFC for prioritizing the candidates with the same priority.

For the datasets at hand related to liver and breast cancer, this led to the detection of a significant set of known disease-associated genes and miRNAs, also introducing a new set of candidates. The topological features introduced via TopControl resulted in a high overlap between the predicted disease-associated genes and the set of known disease-associated genes.

We realized that the topological hotspots identified for one type of tumor overlapped with disease-associated genes and miRNAs that are annotated to other tumors. This indicates the ability of the method in identifying a set of candidates whose abnormal expression can lead to several diseases.

Although we did not find any other tool that works without relying on any prior knowledge, we compared the performance of TopControl with Endeavor for human by providing the minimum amount of required information. TopControl performed equally well on the LIHC dataset and outperformed Endeavor on the BRCA dataset. We noted that the recently updated version of Endeavor does not allow to provide more than 200 candidates for prioritization which reduces the method usability. The potential of TopControl to predict disease-associated genes without relying on any prior knowledge enables the tool to predict the set of candidates for diseases for which few or no disease-associated genes are known.

To compare TopControl top candidates in the fifth layer, we considered two common approaches such as degree and LFC. The same number of candidates sorted either by degree or by LFC were selected for the degree and LFC approaches, respectively. We then checked which of the selected genes and miRNAs are mentioned as disease-associated in DisGeNET and HMDD databases. Considering the results obtained for single features, at thresholds of 25 (LIHC) and 50 (BRCA) and below, more disease-associated genes and miRNAs were identified by the TopControl and degree approaches than by LFC, see Tables S9 and S10. At the highest thresholds (50 for LIHC and 100 for BRCA), all three approaches identified a similar number of disease-associated genes.

Considering the intersection of two features, the overlap between TopControl and degree approaches identified most known disease-associated genes and miRNAs among all the pairwise comparisons for all cases except for the largest threshold. Again, at the highest thresholds (50 for LIHC and 100 for BRCA), all pairwise intersections identified a similar number of disease-associated genes.

## Outlook and Perspective

In addition to well-known features such as LFC and top-degree nodes that are often being used to prioritize candidate disease genes, we showed here that considering the topology of the disease-related gene-regulatory networks may be equally an helpful feature. In this work, we used the TFmiR web server, which integrates genome-wide transcriptional and post-transcriptional regulatory interactions from experimental resources^13^. The quality of the candidates in each layer is reflected by the quality of the candidates in the underlying layers, e.g. the number of hubs and the size of MDS and MCDS that are affected by the density of the underlying network^10,26^. To achieve higher accuracy, we plan to incorporate epigenetic factors for the construction of regulatory networks into TopControl. Moreover, this extension will enable the tool to identify key epigenetic factors when these factors are considered for the construction of the network.

## Methods

In this work, we use a hierarchical model of five layers to prioritize a set of candidates related to a certain disease. Genes in the first layer have the lowest priority to be related to a certain disease and genes placed in the fifth layer have the highest priority, see Fig. 1. Basically, the genes in each layer are a subset of the genes in the lower layer.

In the first layer, TopControl considers the full set of genes whose expression levels between two conditions have been provided. In the second layer, the tool selects the set of genes that are DE between disease and normal conditions. In this work, we used the DESeq method^12^ for the identification of DE genes. We have shown earlier that topological features such as hubs, MDS and MCDS are highly consistent in TF-miRNA co-regulatory networks constructed with the TFmiR web server for four different analysis methods that we tested (DESeq, Voom, edgeR, VST)^27^.

### Candidates in the third layer

As diseases are often caused by rewired interactions between genes at transcriptional and post-transcriptional levels, a differential TF-miRNA co-regulatory network was constructed for the set of DE genes with the TFmiR web server^13^. It includes a set of genes from the second layer that interact with each other and a set of miRNAs. The set of miRNAs was selected such that target genes and regulator TFs of miRNAs are significantly enriched among the input deregulated genes using the hypergeometric test followed by the Benjamini & Hochberg (BH) adjustment^28^ with a cut off p-value of 0.001. The co-regulatory network (termed complete network in TFmiR) includes all the experimentally validated interactions between user-defined DE genes and retrieved miRNAs which are extracted from a variety of regulatory databases^13^. The interaction types are TF → gene, TF → miRNA and miRNA → gene, respectively.

### Candidates in the fourth layer

Genes and miRNAs from the third layer are prioritized if they belong to either MDS, MCDS or hub set. Therefore, hubs, dominators and the nodes in the dominating pathway (we termed the members of a MCDS a dominating pathway) of the underlying TF-miRNA co-regulatory network form the fourth layer. As hub-degree genes and miRNAs, the TFmiR web server outputs the top 10% highest degree nodes^13^. An MDS is calculated based on the ILP formulation described in^10^, where MDS in a regulatory network is the minimum number of nodes that control the whole network. An MCDS is computed based on the heuristic approach mentioned in^10^, where MCDS in a co-regulatory network is a connected set of genes and miRNAs that control the largest connected component (LCC) of the network.

### Candidates in the fifth layer

A score is assigned to the nodes in this layer that is equal to the number of roles they play in the network. The maximum score of three is assigned to a gene or miRNA that is a hub, as well as a dominator and belongs to a dominating pathway of the related network. To select the most promising candidates, TopControl assigns priority to the candidates that are identified by both MDS and MCDS and have high degree of interactions. The top-most candidates in the fifth layer are sorted in descending order based on the absolute value of LFC of expression between two conditions.

### Functional annotation

In this work, the biological function of the genes in the fifth layer was evaluated using the enrichment analysis tool provided at the DAVID portal of NIH (version 6.8) based on the functional categories in GO Direct^29^. *p*-values below the threshold 0.05 obtained by the hypergeometric test were adjusted for multiple testing using the BH method^28^.

## Supplementary information

Supplementary info

## References

[1] Soneson C, Delorenzi M (2013). A comparison of methods for differential expression analysis of RNA-seq data. BMC Bioinformatics.

[2] Ching T, Huang S, Garmire LX (2014). Power analysis and sample size estimation for RNA-Seq differential expression. RNA.

[3] Chen J, Bardes EE, Aronow BJ, Jegga AG (2009). ToppGene Suite for gene list enrichment analysis and candidate gene prioritization. Nucleic Acids Res.

[4] Chen J, Aronow BJ, Jegga AG (2009). Disease candidate gene identification and prioritization using protein interaction networks. BMC Bioinformatics.

[5] Moreau Y, Tranchevent L-CC (2012). Computational tools for prioritizing candidate genes: boosting disease gene discovery. Nature reviews. Genetics.

[6] Tranchevent L-C (2016). Candidate gene prioritization with Endeavour. Nucleic Acids Res.

[7] Kacprowski T, Doncheva NT, Albrecht M (2013). NetworkPrioritizer: A versatile tool for network - based prioritization of candidate disease genes or other molecules. Bioinformatics.

[8] Ernst M (2017). FocusHeuristics-expression-data-driven network optimization and disease gene prediction. Scientific Reports.

[9] Nam, Y., Jhee, J. H., Cho, J., Lee, J.-H. & Shin, H. Disease gene identification based on generic and disease-specific genome networks. *Bioinformatics* bty882 (2018). 10.1093/bioinformatics/bty882 30335143

[10] Nazarieh M, Wiese A, Will T, Hamed M, Helms V (2016). Identification of key player genes in gene regulatory networks. BMC Systems Biology.

[11] Yang W, Rosenstiel PC, Schulenburg H (2016). Absseq: a new rna-seq analysis method based on modelling absolute expression differences. BMC Genomics.

[12] Anders S, Huber W (2010). Differential expression analysis for sequence count data. Genome biology.

[13] Hamed M, Spaniol C, Nazarieh M, Helms V (2015). TFmiR: A web server for constructing and analyzing disease-specific transcription factor and miRNA co-regulatory networks. Nucleic Acids Res.

[14] Nazarieh M, Rajula HSR, Helms V (2019). Topology Consistency of Disease-specific Differential Co-regulatory Networks. BMC bioinformatics.

[15] Bauer-Mehren A, Rautschka M, Sanz F, Furlong LI (2010). DisGeNET: a Cytoscape plugin to visualize, integrate, search and analyze gene-disease networks. Bioinformatics (Oxford, England).

[16] Lu M (2008). An Analysis of Human MicroRNA and Disease Associations. PLoS One.

[17] Chen T (2017). Liverwiki: a wiki-based database for human liver. BMC Bioinformatics.

[18] Will T, Helms V (2016). Ppixpress: construction of condition-specific protein interaction networks based on transcript expression. Bioinformatics.

[19] Weiwei T (2013). Egr-1 enhances drug resistance of breast cancer by modulating mdr1 expression in a ggpps-independent manner. Biomedicine and Pharmacotherapy.

[20] Li X-Q, Lu J-T, Tan C-C, Wang Q-S, Feng Y-M (2016). Runx2 promotes breast cancer bone metastasis by increasing integrin alpha 5 - mediated colonization. Cancer Letters.

[21] Hix LM (2013). Tumor stat1 transcription factor activity enhances breast tumor growth and immune suppression mediated by myeloid-derived suppressor cells. Journal of Biological Chemistry.

[22] Reithmeier A (2017). Tartrate-resistant acid phosphatase (TRAP/ACP5) promotes metastasis-related properties via TGF*β*2/T*β*R and CD44 in MDA-MB-231 breast cancer cells. BMC Cancer.

[23] Schwartz-Roberts JL (2015). Interferon regulatory factor-1 signaling regulates the switch between autophagy and apoptosis to determine breast cancer cell fate. Cancer Res.

[24] Bouafia A (2014). p53 requires the stress sensor usf1 to direct appropriate cell fate decision. PLoS Genetics.

[25] Lee Y (1983). Breast carcinoma: Pattern of metastasis at autopsy. Surgical Oncology.

[26] Wightman P, Fabregas A, Labrador M (2011). A mathematical solution to the mcds problem for topology construction in wireless sensor networks. Latin America Transactions, IEEE (Revista IEEE America Latina).

[27] Nazarieh, M. *Understanding regulatory mechanisms underlying stem cells helps to identify cancer biomarkers*. Ph.D. thesis, Saarland University, Saarbrücken, Germany (2018).

[28] Benjamini Y, Hochberg Y (1995). Controlling the False Discovery Rate: A Practical and Powerful Approach to Multiple Testing. J Roy Statist Soc. Series B (Methodological).

[29] Huang DW, Sherman BT, Lempicki RA (2009). Systematic and integrative analysis of large gene lists using DAVID bioinformatics resources. Nat protocols.

