# Supplementary material for: TopControl: A Tool to Prioritize Candidate Disease-associated Genes based on Topological Network Features
Source: Sci Rep. 2019 Dec 19;9:19472. doi: 10.1038/s41598-019-55954-6 (PMC6923402; doi:10.1038/s41598-019-55954-6)
Supplement: Supplementary file 1 — Supplementary info [file 41598_2019_55954_MOESM1_ESM.pdf]

TopControl: A Tool to Prioritize Candidate Disease-associated Genes based on Topological Network Features

Maryam Nazarieh[1,2,*] and Volkhard Helms[2,*]

[1] *Graduate School of Computer Science, Saarland University, Saarbruecken, Germany*
[2] *Center for Bioinformatics, Saarland University, Saarbruecken, Germany*
[*] *volkhard.helms@bioinformatik.uni-saarland.de,maryam.nazarieh@bioinformatik.uni-saarland.de*

Supplementary material

Table S1: 82 Candidates for hepatocellular carcinoma in the fourth layer identified by TopControl. Genes and miRNAs were sorted initially by their scores, then by LFC. D stands for degree of the node and LFC for $\log_2$(fold change).

gene/miRNA	D	hub	mds	mcds	score	LFC
E2F1	25	1	1	1	3	3.76
EGR1	18	1	1	1	3	2.33
ESR1	9	1	1	1	3	2.19
JUN	33	1	1	1	3	1.39
NR1I2	9	1	1	1	3	1.37
MYC	18	1	1	1	3	1.07
JUND	6	1	1	1	3	0.99
STAT3	10	1	1	1	3	0.81
USF1	15	1	1	1	3	0.73
NR1H4	7	1	1	1	3	0.63
ETS1	9	1	1	1	3	0.61
IRF1	5	1	1	1	3	0.61
NR1I3	8	1	1	1	3	0.6
hsa-let-7b	47	1	1	1	3	-
hsa-mir-26a-5p	16	1	1	1	3	-
hsa-mir-29a	21	1	1	1	3	-
hsa-mir-29a-3p	18	1	1	1	3	-
hsa-mir-34a-5p	28	1	1	1	3	-
POU3F2	2	0	1	1	2	7.44
TP73	4	0	1	1	2	3.92
FOXM1	3	0	1	1	2	3.69
MYCN	3	0	1	1	2	3.25
ETV4	4	0	1	1	2	3.02
FOS	23	1	0	1	2	2.93
IL1B	1	0	1	1	2	2.25
NOS2	5	1	0	1	2	2.2
NR4A1	3	0	1	1	2	2.13
HBB	6	1	0	1	2	2.05
FOXO1	3	0	1	1	2	1.84
CYP3A4	6	1	0	1	2	1.84
IRF8	2	0	1	1	2	1.76
KCNIP3	1	0	1	1	2	1.76
ETS2	5	0	1	1	2	1.68
JUNB	2	0	1	1	2	1.55
FOSL1	10	1	0	1	2	1.37
SATB1	1	0	1	1	2	1.32
CEBPD	1	0	1	1	2	1.3
KLF6	2	0	1	1	2	1.28
KLF4	2	0	1	1	2	1.27
MAFG	3	0	1	1	2	1.21
KLF11	3	0	1	1	2	1.15
PDGFB	1	0	1	1	2	1.11
HIVEP1	1	0	1	1	2	1.04
NME2	1	0	1	1	2	0.95
CYBB	5	1	0	1	2	0.95
TCF3	2	0	1	1	2	0.92
ZBTB7B	1	0	1	1	2	0.88
TNFRSF1A	1	0	1	1	2	0.82
MAZ	4	0	1	1	2	0.8
GATA4	2	0	1	1	2	0.76
F12	1	0	1	1	2	0.75
CREM	3	0	1	1	2	0.74
CNBP	1	0	1	1	2	0.73
FOXA3	1	0	1	1	2	0.7

HLTF	1	0	1	1	2	0.7
SREBF2	3	0	1	1	2	0.68
NFE2L2	4	0	1	1	2	0.66
COL2A1	2	0	0	1	1	10.97
TERT	6	1	0	0	1	9.17
HOXD10	1	0	1	0	1	5.57
OTX1	1	0	1	0	1	5.53
CDK1	2	0	0	1	1	3.41
RRM2	2	0	0	1	1	2.98
SERPINE1	4	0	0	1	1	1.76
HMGA1	5	0	0	1	1	1.65
IGFBP1	2	0	0	1	1	1.45
GTF2IRD1	1	0	1	0	1	1.3
WEE1	1	0	1	0	1	1.25
COL1A2	4	0	0	1	1	1.18
PLAU	8	1	0	0	1	1.1
APOH	6	1	0	0	1	1.09
APOA5	2	0	0	1	1	1.05
ABCA1	2	0	0	1	1	1.02
AR	3	0	1	0	1	1
GATA6	1	0	1	0	1	0.9
HSF1	1	0	1	0	1	0.85
RORA	1	0	1	0	1	0.81
OAS1	1	0	1	0	1	0.8
NFKB2	2	0	1	0	1	0.76
HTATIP2	2	0	0	1	1	0.68
CCND1	12	1	0	0	1	0.67
ALDOC	2	0	0	1	1	0.67

Table S2: Enriched GO terms (top) and KEGG pathways (bottom lines starting with hsa...) with adjusted p-values < 0.05 for the hubs in the hepatocellular carcinoma network. P-values were adjusted for multiple testing using the BH procedure.

Enriched terms	count	*adj. p-values*
GO:0045944 positive regulation of transcription from RNA polymerase II promoter	15	5.138E-10
GO:0045893 positive regulation of transcription, DNA-templated	10	1.316E-6
GO:0006366 transcription from RNA polymerase II promoter	10	1.908E-6
GO:0042493 response to drug	8	1.016E-5
GO:1902895 positive regulation of pri-miRNA transcription from RNA polymerase II promoter	4	2.219E-4
GO:0032355 response to estradiol	5	4.619E-4
GO:0051591 response to cAMP	4	0.001
GO:0032870 cellular response to hormone stimulus	4	0.001
GO:0000122 negative regulation of transcription from RNA polymerase II promoter	8	0.001
GO:0034097 response to cytokine	4	0.002
GO:0048146 positive regulation of fibroblast proliferation	4	0.002
GO:0043401 steroid hormone mediated signaling pathway	4	0.002
GO:0009612 response to mechanical stimulus	4	0.002
GO:0001666 response to hypoxia	5	0.002
GO:0051412 response to corticosterone	3	0.008
GO:0051726 regulation of cell cycle	4	0.017
GO:0045787 positive regulation of cell cycle	3	0.026
GO:0006367 transcription initiation from RNA polymerase II promoter	4	0.027
GO:0030522 intracellular receptor signaling pathway	3	0.029
GO:0007568 aging	4	0.031
GO:0008285 negative regulation of cell proliferation	5	0.037
GO:0042127 regulation of cell proliferation	4	0.039
GO:0042542 response to hydrogen peroxide	3	0.043
GO:0071277 cellular response to calcium ion	3	0.043
GO:0006357 regulation of transcription from RNA polymerase II promoter	5	0.048
hsa05166:HTLV-I infection	9	9.692E-6
hsa04917:Prolactin signaling pathway	5	8.985E-4
hsa05161:Hepatitis B	6	0.001
hsa05200:Pathways in cancer	7	0.006
hsa04380:Osteoclast differentiation	5	0.007
hsa05210:Colorectal cancer	4	0.008
hsa05133:Pertussis	4	0.012
hsa05222:Small cell lung cancer	4	0.015
hsa05205:Proteoglycans in cancer	5	0.016
hsa05206:MicroRNAs in cancer	5	0.043
hsa04310:Wnt signaling pathway	4	0.043
hsa05219:Bladder cancer	3	0.046

Table S3: Enriched GO terms (top) and KEGG pathways (bottom lines) with adjusted p-values < 0.05 for the MDS in the hepatocellular carcinoma network. P-values were adjusted for multiple testing using the BH procedure.

Enriched terms	count	*adj. p-values*
GO:0045944 positive regulation of transcription from RNA polymerase II promoter	43	3.867E-38
GO:0006366 transcription from RNA polymerase II promoter	25	1.062E-19
GO:0045893 positive regulation of transcription, DNA-templated	24	1.923E-18
GO:0000122 negative regulation of transcription from RNA polymerase II promoter	22	6.235E-13
GO:0006351 transcription, DNA-templated	30	1.904E-11
GO:0010628 positive regulation of gene expression	12	9.784E-8
GO:0045892 negative regulation of transcription, DNA-templated	13	6.778E-6
GO:0030522 intracellular receptor signaling pathway	6	1.504E-5
GO:0006367 transcription initiation from RNA polymerase II promoter	8	5.651E-5
GO:0043401 steroid hormone mediated signaling pathway	6	9.578E-5
GO:0051726 regulation of cell cycle	7	2.341E-4
GO:0008285 negative regulation of cell proliferation	10	3.478E-4
GO:0006357 regulation of transcription from RNA polymerase II promoter	10	7.629E-4
GO:0006355 regulation of transcription, DNA-templated	17	9.599E-4
GO:0048146 positive regulation of fibroblast proliferation	5	0.001
GO:0070301 cellular response to hydrogen peroxide	5	0.001
GO:0009612 response to mechanical stimulus	5	0.002
GO:0042493 response to drug	8	0.002
GO:0050728 negative regulation of inflammatory response	5	0.005
GO:0032873 negative regulation of stress-activated MAPK cascade	3	0.006
GO:0045597 positive regulation of cell differentiation	4	0.009
GO:0071499 cellular response to laminar fluid shear stress	3	0.010
GO:0042127 regulation of cell proliferation	6	0.012
GO:2000188 regulation of cholesterol homeostasis	3	0.012
GO:0032870 cellular response to hormone stimulus	4	0.014
GO:0051591 response to cAMP	4	0.014
GO:0045766 positive regulation of angiogenesis	5	0.016
GO:0010941 regulation of cell death	3	0.016
GO:0034097 response to cytokine	4	0.018
GO:0008284 positive regulation of cell proliferation	8	0.021
GO:0010629 negative regulation of gene expression	5	0.027
GO:0060337 type I interferon signaling pathway	4	0.029
GO:0007165 signal transduction	12	0.030
GO:0006006 glucose metabolic process	4	0.033
GO:0045444 fat cell differentiation	4	0.040
GO:1902895 positive regulation of pri-miRNA transcription from RNA polymerase II promoter	3	0.043
GO:0032496 response to lipopolysaccharide	5	0.044
GO:0007568 aging	5	0.044
hsa05166:HTLV-I infection	11	6.294E-5
hsa04010:MAPK signaling pathway	8	0.0181
hsa04380:Osteoclast differentiation	6	0.021

Table S4: Enriched GO terms (top) and KEGG pathways (bottom lines) with adjusted p-values < 0.05 for the MCDS in the hepatocellular carcinoma network. P-values were adjusted for multiple testing using the BH procedure.

Enriched terms	count	*adj. p-values*
GO:0045944 positive regulation of transcription from RNA polymerase II promoter	40	5.582E-30
GO:0006366 transcription from RNA polymerase II promoter	25	3.745E-18
GO:0045893 positive regulation of transcription, DNA-templated	23	1.051E-15
GO:0000122 negative regulation of transcription from RNA polymerase II promoter	19	1.414E-8
GO:0010628 positive regulation of gene expression	12	5.040E-7
GO:0042493 response to drug	12	1.973E-6
GO:0006351 transcription, DNA-templated	25	5.148E-6
GO:0045892 negative regulation of transcription, DNA-templated	13	2.690E-5
GO:0051591 response to cAMP	6	7.307E-5
GO:0009612 response to mechanical stimulus	6	2.315E-4
GO:0006357 regulation of transcription from RNA polymerase II promoter	11	3.759E-4
GO:0008285 negative regulation of cell proliferation	10	0.001
GO:0032870 cellular response to hormone stimulus	5	0.001
GO:0034097 response to cytokine	5	0.002
GO:0048146 positive regulation of fibroblast proliferation	5	0.002
GO:1902895 positive regulation of pri-miRNA transcription from RNA polymerase II promoter	4	0.003
GO:0042127 regulation of cell proliferation	7	0.003
GO:0043401 steroid hormone mediated signaling pathway	5	0.003
GO:0070301 cellular response to hydrogen peroxide	5	0.003
GO:0045766 positive regulation of angiogenesis	6	0.003
GO:0051726 regulation of cell cycle	6	0.004
GO:0010629 negative regulation of gene expression	6	0.006
GO:0032873 negative regulation of stress-activated MAPK cascade	3	0.008
GO:0006367 transcription initiation from RNA polymerase II promoter	6	0.009
GO:0071499 cellular response to laminar fluid shear stress	3	0.013
GO:0030522 intracellular receptor signaling pathway	4	0.013
GO:0007568 aging	6	0.013
GO:0045429 positive regulation of nitric oxide biosynthetic process	4	0.018
GO:0010941 regulation of cell death	3	0.023
GO:0071222 cellular response to lipopolysaccharide	5	0.025
GO:0071277 cellular response to calcium ion	4	0.026
GO:0006915 apoptotic process	9	0.032
GO:0030194 positive regulation of blood coagulation	3	0.033
GO:0008284 positive regulation of cell proliferation	8	0.043
hsa05166:HTLV-I infection	12	3.013E-5
hsa04380:Osteoclast differentiation	8	7.793E-4
hsa05133:Pertussis	6	0.003
hsa05142:Chagas disease (American trypanosomiasis)	6	0.012
hsa04010:MAPK signaling pathway	8	0.020

Table S5: 140 candidates for breast neoplasms in the fourth layer identified by TopControl. They were sorted initially by their scores, then by LFC. D stands for degree of the node and LFC for $\log_2$(fold change).

gene/miRNA	D	hub	mds	mcds	score	LFC
EGR1	19	1	1	1	3	2.59
ESR2	7	1	1	1	3	2.58
FOS	20	1	1	1	3	2.47
E2F1	25	1	1	1	3	2.34
CEBPA	17	1	1	1	3	2.1
ESR1	19	1	1	1	3	1.79
JUN	45	1	1	1	3	1.6
STAT5A	7	1	1	1	3	1.6
RUNX2	5	1	1	1	3	1.4
STAT1	34	1	1	1	3	1.28
ETS2	6	1	1	1	3	1.15
MITF	8	1	1	1	3	0.94
NR1H3	8	1	1	1	3	0.9
TFAP2A	24	1	1	1	3	0.9
IRF1	16	1	1	1	3	0.65
ARHGEF7	11	1	1	1	3	0.62
USF1	16	1	1	1	3	0.61
SRF	5	1	1	1	3	0.58
TFDP1	10	1	1	1	3	0.58
hsa-mir-1	86	1	1	1	3	-
hsa-mir-145-5p	39	1	1	1	3	-
hsa-mir-146a	31	1	1	1	3	-
hsa-mir-21	44	1	1	1	3	-
hsa-mir-21-5p	32	1	1	1	3	-
hsa-mir-34a-5p	27	1	1	1	3	-
EFNA2	2	0	1	1	2	Inf
CGA	5	1	0	1	2	7.25
GBX2	1	0	1	1	2	5.7
GATA4	3	0	1	1	2	5.6
WT1	2	0	1	1	2	5.39
LHX2	1	0	1	1	2	4.88
HBB	5	1	0	1	2	4.73
BMPR1B	1	0	1	1	2	4.41
POU3F2	1	0	1	1	2	4.14
IFNB1	9	1	0	1	2	4.13
RRM2	2	0	1	1	2	3.69
FOXM1	1	0	1	1	2	3.54
KIT	6	1	0	1	2	2.79
IL6	7	1	0	1	2	2.76
HOXA5	2	0	1	1	2	2.55
OTX1	1	0	1	1	2	2.49
TFF3	1	0	1	1	2	2.35
NR4A1	4	0	1	1	2	2.17
TAL1	1	0	1	1	2	1.92
BMP6	1	0	1	1	2	1.87
FOXA1	3	0	1	1	2	1.87
GATA3	3	0	1	1	2	1.75
SOX10	1	0	1	1	2	1.66
IRF7	5	0	1	1	2	1.6
NR3C1	8	1	0	1	2	1.57
PLAU	8	1	0	1	2	1.49
THRB	3	0	1	1	2	1.33
NR5A2	1	0	1	1	2	1.29
LMO2	1	0	1	1	2	1.26
MAZ	4	0	1	1	2	1.24
STAT5B	4	0	1	1	2	1.24

SATB1	1	0	1	1	2	1.16
FLI1	5	0	1	1	2	1.14
JUNB	2	0	1	1	2	1.12
KLF8	1	0	1	1	2	1.11
SERPINE1	7	1	0	1	2	1.11
RARB	6	1	1	0	2	1.1
ETV5	4	0	1	1	2	1.09
TRERF1	1	0	1	1	2	1.08
MYC	21	1	0	1	2	1.07
THRA	2	0	1	1	2	1.07
HEY2	1	0	1	1	2	1.06
NFATC2	3	0	1	1	2	1.06
TNFSF12	3	0	1	1	2	1.05
IRF9	1	0	1	1	2	1.03
MECOM	1	0	1	1	2	1.03
FOXO4	1	0	1	1	2	1.02
SREBF1	7	1	0	1	2	1.02
MEIS1	1	0	1	1	2	1.01
TCF7L2	3	0	1	1	2	1.01
KLF11	4	0	1	1	2	1
NR2F6	2	0	1	1	2	1
CEBPD	4	0	1	1	2	0.97
HMGB2	1	0	1	1	2	0.97
PBX1	2	0	1	1	2	0.93
TEAD4	1	0	1	1	2	0.91
KLF13	2	0	1	1	2	0.88
JUND	5	0	1	1	2	0.87
KRAS	1	0	1	1	2	0.83
RPA3	1	0	1	1	2	0.82
MYEF2	1	0	1	1	2	0.8
TFCP2L1	1	0	1	1	2	0.76
TFE3	1	0	1	1	2	0.68
VEGFA	7	1	0	1	2	0.66
TCF3	3	0	1	1	2	0.65
AR	5	0	1	1	2	0.63
ICAM1	6	1	0	1	2	0.62
MMP1	5	1	0	0	1	6.92
HBG1	4	0	0	1	1	5.42
INSM1	2	0	1	0	1	4.93
APOB	4	0	0	1	1	4.73
ADIPOQ	3	0	0	1	1	4.71
SLC2A4	2	0	0	1	1	4.54
PF4	2	0	0	1	1	4.45
ACACB	1	0	1	0	1	3.71
TYRP1	2	0	0	1	1	3.65
ZBTB16	2	0	0	1	1	3.31
ISG15	4	0	0	1	1	2.95
MUC1	4	0	0	1	1	2.77
CDC25A	5	1	0	0	1	2.32
ALDOC	2	0	0	1	1	2.24
ATF3	6	1	0	0	1	2.21
LEF1	4	0	0	1	1	2.06
PIGR	4	0	0	1	1	2.05
ABCB1	3	0	0	1	1	1.94
ERBB2	6	1	0	0	1	1.89
COL1A2	5	1	0	0	1	1.88
CYP11A1	3	0	0	1	1	1.86
BRCA2	4	0	0	1	1	1.81
EPAS1	1	0	1	0	1	1.73
FOXO1	1	0	1	0	1	1.69
APOC2	2	0	0	1	1	1.5

RFX2	1	0	1	0	1	1.46
ZNF219	1	0	1	0	1	1.43
AFP	1	0	1	0	1	1.43
EGFR	8	1	0	0	1	1.4
PPARA	3	0	0	1	1	1.36
HMGA1	3	0	0	1	1	1.34
PDGFA	2	0	0	1	1	1.33
BRCA1	5	0	0	1	1	1.24
KLF6	1	0	1	0	1	1.24
HDGF	1	0	1	0	1	1.21
PARP1	1	0	1	0	1	1.2
RARA	3	0	1	0	1	1.13
BCL6	3	0	0	1	1	1.09
HOXD9	2	0	1	0	1	1.09
CCND1	15	1	0	0	1	1.01
MYB	6	1	0	0	1	0.99
CCL5	6	1	0	0	1	0.94
SOX4	1	0	1	0	1	0.89
PGR	5	0	0	1	1	0.87
ZEB1	1	0	1	0	1	0.72
ZNF444	1	0	1	0	1	0.65
FUS	2	0	1	0	1	0.64
NR1D1	3	0	1	0	1	0.62

Table S6: Enriched GO terms (top) and KEGG pathways (bottom lines) with adjusted p-values < 0.05 for the hubs in the breast neoplasms network. P-values were adjusted for multiple testing using the BH procedure.

Enriched terms	count	*adj. p-values*
GO:0045944 positive regulation of transcription from RNA polymerase II promoter	27	5.900E-20
GO:0045893 positive regulation of transcription, DNA-templated	16	1.506E-10
GO:0000122 negative regulation of transcription from RNA polymerase II promoter	17	8.232E-10
GO:0006366 transcription from RNA polymerase II promoter	15	1.345E-9
GO:0048661 positive regulation of smooth muscle cell proliferation	6	5.457E-5
GO:0050679 positive regulation of epithelial cell proliferation	6	5.457E-5
GO:0010628 positive regulation of gene expression	8	3.862E-4
GO:0061029 eyelid development in camera-type eye	4	4.089E-4
GO:0045429 positive regulation of nitric oxide biosynthetic process	5	4.226E-4
GO:0048146 positive regulation of fibroblast proliferation	5	6.762E-4
GO:0042493 response to drug	8	6.854E-4
GO:0006357 regulation of transcription from RNA polymerase II promoter	9	7.250E-4
GO:0008284 positive regulation of cell proliferation	9	9.027E-4
GO:0070374 positive regulation of ERK1 and ERK2 cascade	6	0.004
GO:0030335 positive regulation of cell migration	6	0.004
GO:0051091 positive regulation of sequence-specific DNA binding transcription factor activity	5	0.006
GO:0001541 ovarian follicle development	4	0.007
GO:0051591 response to cAMP	4	0.009
GO:0034097 response to cytokine	4	0.013
GO:0008285 negative regulation of cell proliferation	7	0.015
GO:0043406 positive regulation of MAP kinase activity	4	0.017
GO:0060337 type I interferon signaling pathway	4	0.019
GO:0006367 transcription initiation from RNA polymerase II promoter	5	0.020
GO:0035458 cellular response to interferon-beta	3	0.024
GO:0007623 circadian rhythm	4	0.025
GO:0071347 cellular response to interleukin-1	4	0.025
GO:0030324 lung development	4	0.025
GO:0060749 mammary gland alveolus development	3	0.025
GO:0001666 response to hypoxia	5	0.026
GO:0007596 blood coagulation	5	0.029
GO:1902895 positive regulation of pri-miRNA transcription from RNA polymerase II promoter	3	0.031
GO:0045892 negative regulation of transcription, DNA-templated	7	0.032
GO:0046427 positive regulation of JAK-STAT cascade	3	0.035
GO:0007165 signal transduction	10	0.037
GO:0006351 transcription, DNA-templated	13	0.039
GO:0002053 positive regulation of mesenchymal cell proliferation	3	0.045
hsa05200:Pathways in cancer	16	8.489E-8
hsa05166:HTLV-I infection	13	3.355E-7
hsa04917:Prolactin signaling pathway	8	2.475E-6
hsa05219:Bladder cancer	7	2.695E-6
hsa05161:Hepatitis B	9	2.010E-5
hsa05323:Rheumatoid arthritis	7	1.346E-4
hsa05212:Pancreatic cancer	6	4.0173E-4
hsa04620:Toll-like receptor signaling pathway	6	0.002
hsa05142:Chagas disease (American trypanosomiasis)	6	0.002
hsa05221:Acute myeloid leukemia	5	0.003
hsa05223:Non-small cell lung cancer	5	0.003
hsa04151:PI3K-Akt signaling pathway	9	0.004
hsa05205:Proteoglycans in cancer	7	0.006
hsa05206:MicroRNAs in cancer	8	0.006
hsa04630:Jak-STAT signaling pathway	6	0.008
hsa04012:ErbB signaling pathway	5	0.009
hsa04915:Estrogen signaling pathway	5	0.013
hsa04066:HIF-1 signaling pathway	5	0.013
hsa05164:Influenza A	6	0.015
hsa04668:TNF signaling pathway	5	0.015
hsa05213:Endometrial cancer	4	0.015
hsa05168:Herpes simplex infection	6	0.016
hsa05210:Colorectal cancer	4	0.022
hsa05230:Central carbon metabolism in cancer	4	0.022
hsa04110:Cell cycle	5	0.023
hsa04510:Focal adhesion	6	0.023
hsa04380:Osteoclast differentiation	5	0.023
hsa05162:Measles	5	0.024
hsa05160:Hepatitis C	5	0.024
hsa05218:Melanoma	4	0.027
hsa05220:Chronic myeloid leukemia	4	0.027
hsa04060:Cytokine-cytokine receptor interaction	6	0.028
hsa05133:Pertussis	4	0.029
hsa04932:Non-alcoholic fatty liver disease (NAFLD)	5	0.031
hsa05222:Small cell lung cancer	4	0.037
hsa05215:Prostate cancer	4	0.040
hsa05202:Transcriptional misregulation in cancer	5	0.041
hsa05143:African trypanosomiasis	3	0.043
hsa05020:Prion diseases	3	0.043

Table S7: Enriched GO terms (top) and KEGG pathways (bottom lines) with adjusted p-values < 0.05 for the MDS in the breast neoplasms network. P-values were adjusted for multiple testing using the BH procedure.

Enriched terms	count	*adj. p-values*
GO:0045944 positive regulation of transcription from RNA polymerase II promoter	60	7.919E-48
GO:0006366 transcription from RNA polymerase II promoter	39	3.038E-31
GO:0000122 negative regulation of transcription from RNA polymerase II promoter	38	1.036E-24
GO:0045893 positive regulation of transcription, DNA-templated	33	1.777E-23
GO:0006351 transcription, DNA-templated	44	3.246E-15
GO:0006357 regulation of transcription from RNA polymerase II promoter	22	2.381E-12
GO:0045892 negative regulation of transcription, DNA-templated	22	2.294E-11
GO:0006367 transcription initiation from RNA polymerase II promoter	14	2.058E-10
GO:0043401 steroid hormone mediated signaling pathway	10	2.195E-9
GO:0030522 intracellular receptor signaling pathway	8	1.236E-7
GO:0006355 regulation of transcription, DNA-templated	29	2.346E-7
GO:0008285 negative regulation of cell proliferation	16	2.543E-7
GO:0045597 positive regulation of cell differentiation	7	3.505E-6
GO:0034097 response to cytokine	7	2.672E-5
GO:0045666 positive regulation of neuron differentiation	7	2.814E-4
GO:0045669 positive regulation of osteoblast differentiation	6	0.001
GO:0001938 positive regulation of endothelial cell proliferation	6	0.002
GO:0042493 response to drug	10	0.002
GO:0003215 cardiac right ventricle morphogenesis	4	0.002
GO:0048469 cell maturation	5	0.002
GO:0035855 megakaryocyte development	4	0.004
GO:0032870 cellular response to hormone stimulus	5	0.004
GO:0051591 response to cAMP	5	0.005
GO:0071277 cellular response to calcium ion	5	0.007
GO:0009612 response to mechanical stimulus	5	0.012
GO:0060337 type I interferon signaling pathway	5	0.016
GO:0042127 regulation of cell proliferation	7	0.019
GO:0071773 cellular response to BMP stimulus	4	0.020
GO:0051726 regulation of cell cycle	6	0.021
GO:0045444 fat cell differentiation	5	0.023
GO:0045647 negative regulation of erythrocyte differentiation	3	0.035
GO:0043065 positive regulation of apoptotic process	8	0.039
GO:0030218 erythrocyte differentiation	4	0.041
GO:0033148 positive regulation of intracellular estrogen receptor signaling pathway	3	0.041
GO:0048646 anatomical structure formation involved in morphogenesis	3	0.047
GO:0010941 regulation of cell death	3	0.047
GO:0008584 male gonad development	5	0.049
hsa05202:Transcriptional misregulation in cancer	13	1.158E-6
hsa05200:Pathways in cancer	16	3.003E-5
hsa04917:Prolactin signaling pathway	8	7.036E-5
hsa05161:Hepatitis B	9	7.276E-4
hsa05166:HTLV-I infection	11	0.001
hsa04380:Osteoclast differentiation	8	0.001
hsa05221:Acute myeloid leukemia	6	0.001
hsa04919:Thyroid hormone signaling pathway	7	0.005
hsa05220:Chronic myeloid leukemia	5	0.039

Table S8: Enriched GO terms (top) and KEGG pathways (bottom lines) with adjusted p-values < 0.05 for the MCDS in the breast neoplasms network. P-values were adjusted for multiple testing using the BH procedure.

Enriched terms	count	*adj. p-values*
GO:0045944 positive regulation of transcription from RNA polymerase II promoter	66	5.561E-50
GO:0006366 transcription from RNA polymerase II promoter	40	3.410E-29
GO:0045893 positive regulation of transcription, DNA-templated	36	2.942E-24
GO:0000122 negative regulation of transcription from RNA polymerase II promoter	35	1.772E-18
GO:0006357 regulation of transcription from RNA polymerase II promoter	22	1.264E-10
GO:0045892 negative regulation of transcription, DNA-templated	23	1.268E-10
GO:0006367 transcription initiation from RNA polymerase II promoter	14	2.797E-9
GO:0043401 steroid hormone mediated signaling pathway	10	1.531E-8
GO:0030522 intracellular receptor signaling pathway	8	6.067E-7
GO:0006351 transcription, DNA-templated	36	7.362E-7
GO:0042127 regulation of cell proliferation	12	3.413E-6
GO:0042493 response to drug	14	7.937E-6
GO:0010628 positive regulation of gene expression	13	1.161E-5
GO:0008285 negative regulation of cell proliferation	15	2.143E-5
GO:0006355 regulation of transcription, DNA-templated	28	4.631E-5
GO:0045669 positive regulation of osteoblast differentiation	7	2.024E-4
GO:0035855 megakaryocyte development	5	2.355E-4
GO:0060337 type I interferon signaling pathway	7	2.640E-4
GO:0045597 positive regulation of cell differentiation	6	2.768E-4
GO:0001938 positive regulation of endothelial cell proliferation	7	3.703E-4
GO:0032870 cellular response to hormone stimulus	6	6.743E-4
GO:0051591 response to cAMP	6	7.186E-4
GO:0034097 response to cytokine	6	0.001
GO:0008284 positive regulation of cell proliferation	13	0.002
GO:0042593 glucose homeostasis	7	0.002
GO:0051091 positive regulation of sequence-specific DNA binding transcription factor activity	7	0.003
GO:0071356 cellular response to tumor necrosis factor	7	0.003
GO:0048469 cell maturation	5	0.004
GO:0001666 response to hypoxia	8	0.005
GO:0035162 embryonic hemopoiesis	4	0.006
GO:0006978 DNA damage response, signal transduction by p53 class mediator resulting in transcription of p21 class mediator	4	0.006
GO:0060749 mammary gland alveolus development	4	0.007
GO:0001701 in utero embryonic development	8	0.008
GO:0032332 positive regulation of chondrocyte differentiation	4	0.010
GO:0030318 melanocyte differentiation	4	0.011
GO:0008584 male gonad development	6	0.014
GO:0048146 positive regulation of fibroblast proliferation	5	0.015
GO:0045648 positive regulation of erythrocyte differentiation	4	0.018
GO:0030878 thyroid gland development	4	0.020
GO:0048589 developmental growth	4	0.020
GO:0030097 hemopoiesis	5	0.020
GO:0009612 response to mechanical stimulus	5	0.020
GO:0002053 positive regulation of mesenchymal cell proliferation	4	0.021
GO:0032496 response to lipopolysaccharide	7	0.022
GO:0045931 positive regulation of mitotic cell cycle	4	0.026
GO:0001569 patterning of blood vessels	4	0.026
GO:0071773 cellular response to BMP stimulus	4	0.031
GO:0030855 epithelial cell differentiation	5	0.034
GO:0060333 interferon-gamma-mediated signaling pathway	5	0.035
GO:0045444 fat cell differentiation	5	0.036
GO:0007596 blood coagulation	7	0.036
GO:0051726 regulation of cell cycle	6	0.036
GO:1902042 negative regulation of extrinsic apoptotic signaling pathway via death domain receptors	4	0.036
GO:0007623 circadian rhythm	5	0.039
GO:0030501 positive regulation of bone mineralization	4	0.039
GO:0032869 cellular response to insulin stimulus	5	0.040
GO:0045647 negative regulation of erythrocyte differentiation	3	0.040
GO:0030509 BMP signaling pathway	5	0.040
GO:0045666 positive regulation of neuron differentiation	5	0.041
hsa05202:Transcriptional misregulation in cancer	16	5.609E-8
hsa05200:Pathways in cancer	20	3.179E-6
hsa05221:Acute myeloid leukemia	9	8.284E-6
hsa05161:Hepatitis B	12	2.050E-5
hsa05166:HTLV-I infection	15	2.333E-5
hsa04917:Prolactin signaling pathway	9	2.708E-5
hsa04380:Osteoclast differentiation	9	0.002
hsa05210:Colorectal cancer	6	0.011
hsa05160:Hepatitis C	8	0.011
hsa05220:Chronic myeloid leukemia	6	0.018
hsa04919:Thyroid hormone signaling pathway	7	0.023
hsa05215:Prostate cancer	6	0.036
hsa05162:Measles	7	0.041
hsa05216:Thyroid cancer	4	0.042

Table S9: Number of identified disease-associated genes and miRNAs by TopControl, degree and LFC. TopControl considers score 3 in the fifth layer (thresholds 10, 18 for LIHC and 10, 25 for BRCA datasets) and greater than 1 (thresholds 25, 50 for LIHC and 50, 100 for BRCA datasets). TD = intersect (TopControl , degree), TL = intersect(TopControl , LFC), DL = intersect(degree , LFC), TDL = intersect(TopControl , degree, LFC)

Dataset	Threshold	TopControl	Degree	LFC	TD	TL	DL	TDL
LIHC	10	4	6	3	3	1	1	1
LIHC	18	6	8	6	6	2	3	2
LIHC	25	8	9	6	7	4	4	3
LIHC	50	10	13	11	8	8	8	6
BRCA	10	6	5	1	2	0	0	0
BRCA	25	10	9	4	7	0	1	0
BRCA	50	16	18	12	13	10	8	7
BRCA	100	23	25	24	18	17	18	13

Table S10: Identified disease-associated-genes and miRNAs by TopControl, degree and LFC.

LIHC-10	TopControl = E2F1, ESR1, JUN, MYC<br>Degree = hsa-let-7b, JUN, E2F1, FOS, hsa-mir-29a, MYC<br>LFC = TERT, E2F1, FOXM1
LIHC-18	TopControl = E2F1, ESR1, JUN, MYC, hsa-let-7b, hsa-mir-29a<br>Degree = hsa-let-7b, JUN, E2F1, FOS, hsa-mir-29a, MYC, CCND1, ESR1<br>LFC = TERT, E2F1, FOXM1, RRM2, FOS, ESR1
LIHC-25	TopControl = E2F1, ESR1, JUN, MYC, hsa-let-7b, hsa-mir-29a, FOXM1, FOS<br>Degree = hsa-let-7b, JUN, E2F1, FOS, hsa-mir-29a, MYC, CCND1, ESR1, TERT<br>LFC = TERT, E2F1, FOXM1, RRM2, FOS, ESR1
LIHC-50	TopControl = E2F1, ESR1, JUN, MYC, hsa-let-7b, hsa-mir-29a, FOXM1, FOS, CEBPD, PDGFB<br>Degree = hsa-let-7b, JUN, E2F1, FOS, hsa-mir-29a, MYC, CCND1, ESR1, TERT, NFE2L2, FOXM1, SREBF2, AR<br>LFC = TERT, E2F1, FOXM1, RRM2, FOS, ESR1, JUN, CEBPD, PDGFB, MYC, AR
BRCA-10	TopControl = ESR2, FOS, E2F1, ESR1, JUN, STAT5A<br>Degree = JUN, hsa-mir-21, hsa-mir-146a, E2F1, TFAP2A<br>LFC = WT1
BRCA-25	TopControl = ESR2, FOS, E2F1, ESR1, JUN, STAT5A, ETS2, TFAP2A, hsa-mir-146a, hsa-mir-21<br>Degree = JUN, hsa-mir-21, hsa-mir-146a, E2F1, TFAP2A, FOS, ESR1, CCND1, IFNB1<br>LFC = WT1, IFNB1, FOXM1, KIT
BRCA-50	TopControl = ESR2, FOS, E2F1, ESR1, JUN, STAT5A, ETS2, TFAP2A, hsa-mir-146a, hsa-mir-21, WT1, IFNB1, FOXM1, KIT, IL6, FOXA1<br>Degree = JUN, hsa-mir-21, hsa-mir-146a, E2F1, TFAP2A, FOS, ESR1, CCND1, IFNB1, EGFR, ESR2, STAT5A, IL6, ETS2, KIT, RARB, ERBB2, AR<br>LFC = WT1, IFNB1, FOXM1, KIT, IL6, ESR2, FOS, E2F1, ERBB2, FOXA1, BRCA2, ESR1
BRCA-100	TopControl = ESR2, FOS, E2F1, ESR1, JUN, STAT5A, ETS2, TFAP2A, hsa-mir-146a, hsa-mir-21, WT1, IFNB1, FOXM1, KIT, IL6, FOXA1, RARB, TRERF1, HEY2, MEIS1, NR2F6, KRAS, AR<br>Degree = JUN, hsa-mir-21, hsa-mir-146a, E2F1, TFAP2A, FOS, ESR1, CCND1, IFNB1, EGFR, ESR2, STAT5A, IL6, ETS2, KIT, RARB, ERBB2, AR, BRCA1, PGR, BRCA2, FOXA1, WT1, NR2F6, PDGFA<br>LFC = WT1, IFNB1, FOXM1, KIT, IL6, ESR2, FOS, E2F1, ERBB2, FOXA1, BRCA2, ESR1, JUN, STAT5A, AFP, EGFR, PDGFA, BRCA1, PARP1, ETS2, RARB, TRERF1, HEY2, MEIS1
